# *APOE ε4* and ischemic heart disease in American Indian/Indigenous tribal Elders

**DOI:** 10.1016/j.ahjo.2025.100662

**Published:** 2025-11-04

**Authors:** Rene Labounek, Matti J. Matheson, Ashley J. Petersen, Adam Hansen, Adam D. Block, Corey Strong, Annamarie Hill, Meghan Kremer, Ann J. Robertson, Valmiki Maharaj, Kamakshi Lakshminaryan, Danni Li, J. Neil Henderson, Igor Nestrasil, Christophe Lenglet, William G. Mantyh

**Affiliations:** a Division of Clinical Behavioral Neuroscience, Department of Pediatrics, University of Minnesota, Minneapolis, MN, 55414, United States of America; b Department of Neurology, University of Minnesota, Minneapolis, MN, 55455, United States of America; c Division of Biostatistics, School of Public Health, University of Minnesota, Minneapolis, MN, 55455, United States of America; d Bois Forte Band of Chippewa, Health Services, Nett Lake, MN, 55771, United States of America; e Minnesota Department of Health, St. Paul, MN, 55164, United States of America; f Community Outreach, Strategy, External Relations, University of Minnesota, United States of America; g Department of Laboratory Medicine and Pathology, University of Minnesota, Minneapolis, MN, 55455, United States of America; h Department of Medicine, Division of Cardiology, University of Minnesota, Minneapolis, MN, 55455, United States of America; i Department of Family Medicine and Biobehavioral Health, University of Minnesota Duluth, Duluth, MN, 55805, United States of America; j Center for Magnetic Resonance Research, University of Minnesota, Minneapolis, MN, 55455, United States of America

**Keywords:** *APOE ε4*, Ischemic heart disease, Hyperlipidemia, Hypertension, American Indian, Indigenous

## Abstract

**Objective:**

American Indian/Indigenous (AI) populations have the highest rate of ischemic heart disease (IHD) of any racial or ethnic group in the United States. While modifiable cardiovascular risk factors represent a well-established source of elevated IHD in AI, little is known regarding genetic IHD influences, in particular *APOE ε4*, which has an ancestry-dependent prevalence and effect on human disease. We sought to quantify the prevalence and association between *APOE ε4* and IHD in AI communities.

**Methods:**

We performed a cross-sectional, community-based study including tribal Elders (ages >54 years) at the Bois Forte Band of Chippewa in the state of Minnesota. We collected data pertaining to demographics, cardiovascular risk factors, *APOE ε4* genotype, and ischemic heart disease (defined as history of myocardial infarction, coronary artery bypass graft, angiogram showing coronary artery disease, percutaneous transluminal coronary angioplasty, or thrombolytic therapy).

**Findings:**

One-hundred-eighty-one participants were included. Their median age and interquartile range were 67 (61, 73) years, AI ancestry was 75 % (50 %, 100 %), 126 (70 %) were females, 46 (25 %) were *APOE ε4* heterozygous, and 5 (2.8 %) were *APOE ε4* homozygous. Each *APOE ε4* allele increased the odds of IHD in AI tribal Elders 2.38-fold (95 % CI: 0.94–6.89; *p* = 0.06), which is comparable to a two-point rise in hemoglobin A1C.

**Conclusions:**

*APOE ε4* appears to play an important role in the risk of IHD in AI populations, given its high prevalence and strong association with IHD.

## 1 Introduction

Ischemic heart disease (IHD) is the leading cause of mortality both in the United States and globally [1,2]. American Indian/Indigenous (AI) populations face some of the highest rates of IHD of any population, which has been ascribed to elevated rates of cardiometabolic risk factors, most notably type 2 diabetes mellitus (T2DM), hypertension, and social determinants of health that disproportionately affect AI communities [[3], [4], [5], [6]]. The genetic contribution to IHD in AI communities, however, is a relatively understudied area. The Strong Heart Study [7] is the largest AI biomedical study that includes 11 Tribal Nations across 3 geographic regions in the United States. This study demonstrated that *APOE ε4*, the most significant genetic risk factor for Alzheimer's disease and also a risk factor for IHD [8], worsens lipid and lipoprotein profiles (e.g., *APOE ε4* is associated with elevated LDL-C, lower HDL—C, elevated ApoB, and lower ApoA-I). This study did not, however, examine *APOE ε4*'s independent impact on IHD [9]. Determining the effects of *APOE ε4* on IHD in AI populations is complicated by several factors. Firstly, the frequency of *APOE ε4* appears to be inversely correlated with the degree of AI ancestry, with the implication that *APOE ε4* may be too uncommon to significantly increase the population-level risk of IHD, at least in AI populations with a high degree of AI ancestry [10,11]. Secondly, AI communities generally face a high burden of cardiometabolic risk factors, most notably T2DM, that dramatically increase the risk of IHD, which may eclipse contributions from genetic risk factors such as *APOE ε4* [4].

Extrapolating *APOE ε4*-IHD risk from the general US population data to AI communities is not possible due to *APOE ε4*'s ancestry-dependent risk on human disease. In the case of Alzheimer's disease, more than a factor of 10 separates Japanese versus Caribbean Hispanics for *APOE ε4* homozygotes' associated risk of AD [12,13]. AI ancestry may protect against the deleterious effects of *APOE ε4*: recent work has demonstrated an attenuated effect of *APOE ε4* on the risk of cognitive decline, dementia, and radiographic features of Alzheimer's disease in AI populations [10,11,14]. While the literature is not yet well-established, there may be similar ancestry-related differences in *APOE ε4*'s link to IHD. *APOE ε4* independently increases the risk of IHD in British [15], Han Chinese [16,17], Pakistani [18], and white American populations. In contrast, in Taiwanese [19], Omani [20], Alaska Native [21] and Afro-Caribbean [22] populations, *APOE ε4* only indirectly increases IHD risk through hyperlipidemia, but has no detectable independent contributions to IHD. *APOE ε4* is commonly and increasingly used for personalized medicine, as a direct-to-consumer test, and is also now a required part of risk counseling for amyloid-beta monoclonal antibodies for Alzheimer's disease [23]. Better information about the contributions to IHD in AI communities, such as *APOE ε4*, is a required step to designing effective interventions for decreasing the heavy burden of IHD in AI communities. The present AI-focused study, originally designed to examine dementia risk factors, including IHD and *APOE ε4*, allows us to measure *APOE ε4*'s impact on IHD in an AI population of the upper Midwest centered in Minnesota, USA.

## 2 Methods

### 2.1 Standard protocol approvals, registrations, and patient consents

Each participant (or their legally authorized representative if cognitive loss was to an extent that precluded informed consent) provided written informed consent to participate in this cross-sectional study. The Bois Forte Band of Chippewa partnered with the research team, approved this work through a signed, unanimously agreed-on tribal resolution, and approved the publication and public dissemination of this article. The University of Minnesota Institutional Review Board also approved this study.

### 2.2 Participants and study procedures

Participants were recruited via flyers posted on local bulletin boards and social media, health fair booths and presentations, the study team's website, and tribal radio advertisements. These advertisements noted that researchers hoped to include AI participants in a study of cognitive aging clinical research. Participants had to meet the following criteria to be eligible for the study: (1) currently enrolled member of a federally recognized American Indian Tribal Nation, (2) aged >54 years, (3) speak English as a first language, and (4) have no unstable medical conditions requiring frequent hospitalization.

Each participant underwent a standard cognitive neurology History & Physical with the study's subspecialty-trained behavioral neurologist and principal investigator (W.G.M.) or a behavioral neurology specialty-trained nurse practitioner (M.M.). The study collected demographic information, including each participant's age, sex, body mass index (BMI), and percentage of Native ancestry. Percent Native ancestry was based on self-reported “blood quantum”, a flawed but rigorously kept historical record of AI ancestry dating back to the 18th and 19th centuries, which has major implications for treaty rights, self-identity, and tribal enrollment [24]. Each participant was asked whether they had “high cholesterol, diabetes, high blood pressure, chronic kidney disease, or heart disease”. For those who provided a blood sample, we obtained a standard lipid panel, hemoglobin A1C (HbA1C) test, and estimated glomerular filtration rate (eGFR). Low-density lipoprotein cholesterol (LDL-C) was calculated from the standard lipid panel using Eq. (2) of Sampson et al. (2020) [25], which is more accurate for those with hypertriglyceridemia and/or low LDL-C than the commonly used Friedewald or Martin equations. *APOE* genotyping was performed using standard procedures as described by Hixson and Vernier [26]. In brief, genomic DNA was amplified by PCR, followed by restriction digestion with Hhal. Resultant fragments were separated on a high-resolution agarose gel.

### 2.3 Statistical analysis

Between-group differences were assessed with the Wilcoxon rank sum test, Pearson's Chi-squared test, or Fisher's exact test. Tests with *p* < 0.05 identified variables that demonstrated statistically significant between-group differences.

The statistical analysis aimed to explain the outcome of interest as a linear mixture of the predictor of interest and other covariates. The ***outcome of interest*** was a self-reported previous diagnosis of IHD. Each participant was asked whether they had any history of heart disease. If yes, they were asked to describe their heart condition. IHD was classified as present if any of the following were reported: myocardial infarction, history of coronary artery bypass graft, angiogram showing coronary artery disease, percutaneous transluminal coronary angioplasty, or thrombolytic therapy. If they were unsure or there was insufficient information, they were classified as not otherwise specified (NOS). For this analysis, those with NOS heart disease were considered to be non-ischemic given the distinct clinical symptoms indicative of IHD (hospitalization for myocardial infarction, cardiothoracic surgery for coronary artery bypass grafting). The ***predictor of interest*** was the number of *APOE ε4* alleles: zero (genotypes: *ε*2*ε*2, *ε*2*ε*3, *ε*3*ε*3), one (*ε*2*ε*4, *ε*3*ε*4), or two (*ε*4*ε*4).

To estimate the association between *APOE ε4* alleles and IHD, L2-penalized (ridge) logistic regression with the outcome of IHD and an unpenalized predictor of the number of *APOE ε4* alleles was used with two sets of adjustment covariates. The first model included the adjustment covariates of age, sex, LDL-C, and HbA1C, while the second model additionally included indicator variables for self-reported hypertension, self-reported hyperlipidemia, eGFR < 60 mL/min/1.73m^2^, and statin use. In both models, the tuning parameter was chosen to minimize the deviance using 10-fold cross-validation to avoid overfitting the data. The effect of *APOE ε4* was modeled linearly as the number of alleles, since there were too few homozygotes to model it categorically. The L2-penalized logistic regression was used in lieu of standard logistic regression due to the small number of events (IHD) relative to the number of adjustment covariates; however, the *APOE ε4* predictor was not penalized to obtain an unbiased estimate [27]. A 95 % confidence interval (CI) for the *APOE ε4* log odds ratio was constructed using the nonparametric percentile bootstrap, with the *p*-value calculated by inverting the CI. The tuning parameter was reselected in each bootstrap sample. To compare the effect sizes between *APOE ε4* and HbA1C, the secondary model described above was refit, with the unpenalized predictor switched to HbA1C. All analyses were performed in R version 4.2.1 (R Foundation for Statistical Computing, Vienna, Austria) using the *glmnet* [28] and *boot* [29,30] R packages.

## 3 Results

Between October 2021 and March 2025, 297 (204/69 % females) AI Elders had a clinician visit for this study. Two hundred eighty-eight individuals consented to have blood drawn for *APOE* genetic testing, which occurred at a future appointment. Blood draws were obtained from 184 of the consented participants for *APOE* genotyping. An additional 3 participants with blood draws were excluded as they did not have HbA1C measured, leaving a total of 181 participants included in the present analysis. Supplementary Table 1 compares the characteristics of those included in the analysis vs. those excluded due to lack of a blood sample, genotyping, or HbA1C. Similar proportions were included by IHD status (IHD: 63 %, no IHD: 61 %). When stratified by IHD status, participant characteristics were comparable between those included vs. excluded from the analysis, except for age. Those without IHD included in the analysis tended to be older than those excluded (median age, 67 vs. 64; *p* = 0.03). However, among those included in the analysis, the age distributions of those with IHD and those without were similar.

Of the 181 participants in the analysis, 25 (14 %) had IHD. Table 1 summarizes the participant characteristics and compares them by IHD status. Overall, participants had a median age of 67, 70 % were female, and the median percentage of Native American ancestry was 75 %. The most common *APOE* genotypes were *ε*3*ε*3 (67 %) and *ε*3*ε*4 (24 %). Other health conditions were more prevalent among those with IHD (hypertension: 88 % vs. 55 %; hyperlipidemia: 96 % vs. 46 %; chronic kidney disease: 24 % vs. 9.0 %). HbA1C tended to be higher for those with IHD, while LDL-C was lower in those with IHD. In particular, median LDL-C was highest among those with no reported IHD or hyperlipidemia (*n* = 84). This group reported minimal statin use (2.4 %) despite 13 % having LDL-C > 130. In comparison, 75 % of those with IHD and hyperlipidemia (*n* = 24) reported statin use, and only 4.2 % had LDL-C > 130. Among those with hyperlipidemia and no IHD (*n* = 72), 51 % reported statin use, and only 5.4 % on statins had LDL-C > 130, compared to 26 % not on statins. (Only one person reported having IHD and no hyperlipidemia.)

**Table 1:** Study population characteristics by ischemic heart disease status.

Characteristic	N	Overall^a^ *N* = 181	No ischemic heart disease^a^ *N* = 156	Ischemic heart disease^a^ *N* = 25	p-Value^b^
Age [years]	181	67 (61, 73)	67 (61, 72)	67 (63, 76)	0.18
Female	181	126 (70 %)	112 (72 %)	14 (56 %)	0.11
Body mass index [kg/m^2^]	154	30 (26, 35)	30 (26, 34)	32 (26, 35)	0.27
Percentage of native ancestry	134	75 (50, 100)	75 (50, 100)	89 (65, 100)	0.38
*APOE* genotype	181				0.26
ε2ε3		8 (4.4 %)	7 (4.5 %)	1 (4.0 %)	
ε2ε4		2 (1.1 %)	2 (1.3 %)	0 (0 %)	
ε3ε3		122 (67 %)	108 (69 %)	14 (56 %)	
ε3ε4		44 (24 %)	36 (23 %)	8 (32 %)	
ε4ε4		5 (2.8 %)	3 (1.9 %)	2 (8.0 %)	
Tobacco use	180				0.76
Current		75 (42 %)	66 (43 %)	9 (36 %)	
Former		36 (20 %)	30 (19 %)	6 (24 %)	
Never		69 (38 %)	59 (38 %)	10 (40 %)	
Alcohol use	180				0.94
Current		41 (23 %)	36 (23 %)	5 (20 %)	
Former		76 (42 %)	65 (42 %)	11 (44 %)	
Never		63 (35 %)	54 (35 %)	9 (36 %)	
Participant exercises	152	100 (66 %)	86 (67 %)	14 (61 %)	0.59
Self-reported hypertension	181	108 (60 %)	86 (55 %)	22 (88 %)	0.002
Self-reported hyperlipidemia	181	96 (53 %)	72 (46 %)	24 (96 %)	<0.001
Self-reported chronic kidney disease	181	20 (11 %)	14 (9.0 %)	6 (24 %)	0.04
Hemoglobin A1C	181	6.1 (5.7, 7.1)	6.0 (5.7, 7.1)	6.7 (6.1, 8.1)	0.01
Cholesterol	181	165 (134, 196)	167 (138, 199)	148 (129, 189)	0.06
HDL-C	181	48 (41, 62)	48 (41, 63)	47 (42, 54)	0.29
LDL-C	181	82 (60, 112)	86 (63, 113)	60 (56, 87)	0.01
Triglycerides	181	140 (103, 205)	139 (100, 197)	192 (111, 253)	0.13
Glomerular filtration rate^c^ < 60	180	28 (16 %)	23 (15 %)	5 (20 %)	0.55
Statin use	181	57 (31 %)	39 (25 %)	18 (72 %)	<0.001

a Median (IQR); n (%).

b Wilcoxon rank sum test; Pearson's Chi-squared test; Fisher's exact test.

c Units are mL/min/1.73m^2^.

Table 2 summarizes the odds ratios of IHD for the predictors in the investigated L2-penalized logistic regression models. For the model adjusting for age, sex, HbA1C, and LDL-C, the odds of IHD were 2.14 times greater (95 % CI: 0.90–4.76) for each additional *APOE ε4* allele. Similarly, for the model that additionally adjusted for hypertension, hyperlipidemia, low eGFR, and statin use, the odds of IHD were 2.38 times greater (95 % CI: 0.94–6.89) for each additional *APOE ε4* allele. In the comparison model with unpenalized HbA1C, the odds of ICH were 1.35 times greater (95 % CI: 0.63–2.53) for each 2-point increase in HbA1C.

**Table 2:** Odds ratios of ischemic heart disease, estimated from L2-penalized logistic regression models where the primary predictor of *APOE ε4* was unpenalized in the first two models and hemoglobin A1C (HbA1C) was unpenalized in the last model. We only report 95 % confidence intervals (CIs) for the unpenalized predictor, as the penalized predictors are intentionally estimated with bias in L2-penalized logistic regression models to avoid overfitting the data.

Predictor	Primary model: Odds ratio	Secondary model: Odds ratio	HbA1C model: Odds ratio
*APOE ε4*	2.14 (95 % CI: 0.90, 4.76); *p* = 0.08	2.38 (95 % CI: 0.94, 6.89); p = 0.06	1.86
Age	1.04	1.03	1.03
Female	0.53	0.60	0.60
Hemoglobin A1C (2-point increase)	1.59	1.24	1.35 (95 % CI: 0.63, 2.53); *p* = 0.37
LDL-C	0.99	0.99	0.99
Hypertension	–	1.74	1.75
Hyperlipidemia	–	3.22	3.08
Estimated glomerular filtration rate^a^ < 60	–	1.05	1.03
Statin use	–	2.18	2.16

a Units are mL/min/1.73m^2^.

## 4 Discussion

Our study demonstrates that, despite the attenuated association between *APOE ε4* and Alzheimer's disease seen in AI, *APOE ε4* strongly associates with IHD in AI tribal Elders, even independently of hyperlipidemia [15,31]. *APOE ε4* is also common; 28 % of our participants were *APOE ε4* carriers. This stands in contrast to the low rate (9–12 %) of *APOE ε4* carrier status in AI participants with high degrees of AI ancestry who were part of the Choctaw Nation of Oklahoma and Cherokee Nation [[7], [8], [9], [10], [11]]. Our data suggest *APOE ε4* is common and plays a large role in increasing IHD in AI populations, which is on par with the well-known risk posed by T2DM.

Paradoxically, LDL was lower in participants with IHD. We presume that this is due to reverse causality, where participants who suffered IHD were more likely to seek medical attention and be started on high-intensity statin therapy to lower LDL. A self-reported history of hyperlipidemia was associated with IHD, which, following the same logic, may be because patients with IHD found out during their contact with a medical provider that they also had high cholesterol. Supporting this theory are two factors: 1) there was a paradoxical inverse relationship between hyperlipidemia status and LDL, where patients with self-reported hyperlipidemia had lower LDL levels; 2) there was a higher proportion of participants on statin therapy in participants with self-reported hyperlipidemia.

Limitations of the current study are numerous. Firstly, there is enormous inter- and intra-tribal heterogeneity among the 574 federally recognized tribal nations, which precludes generalization of this study across all AI populations. Secondly, as we only enrolled participants who are older than 54, *APOE ε4* carriers with IHD may have passed away at an earlier age, which would bias our study towards underestimating the association between *APOE ε4* and IHD. The recruiting age threshold may explain the larger number of recruited women (68 %), as the life expectancy of AI males is the lowest of any racial category in the US. [32,33] Moreover, the overall AI life expectancy dropped even more post-COVID19 pandemic when compared to the non-Hispanic white sample. [[34], [35], [36]] Thirdly, our study suffered from a significant number of participants who did not complete a blood draw. The blood draw was scheduled on a different date for logistical reasons. However, many of our participants lacked a phone or a reliable method of communication, or had cultural-based reservations regarding blood draws, resulting in many participants who did not complete the blood draw portion of our research, which in turn could introduce a selection bias. We note, however, that the comparison of demographics and medical history was similar between the group who completed versus did not complete the blood draw. Fourthly, as with any study including participant-reported data, recall bias for prior health history is inherent. Finally, our sample size, while large in terms of the representation of AI participants compared to the general dearth of AI representation in the medical literature, is small compared to more recent studies exploring *APOE ε4* and IHD. Larger studies would not only offer a more precise estimate of the *APOE ε4*-IHD relationship, but also allow the additional study of gene-environment interactions, such as the effects of air pollution, [37] post-traumatic stress syndrome, [38] exercise habits, [39] ultra-processed foods, [40] and other environmental risk factors that may detrimentally synergize with *APOE ε4*. In conclusion, *APOE ε4* is highly prevalent and likely plays a large role in the heavy burden IHD AI communities face.

The following is the supplementary data related to this article.

Supplementary Table 1 Comparison of participant characteristics of those included in the analysis vs. those excluded due to no blood sample, genotyping, or hemoglobin A1C (HbA1C). Supplementary Table 1

## CRediT authorship contribution statement

**Rene Labounek:** Data curation, Formal analysis, Methodology, Writing – original draft, Writing – review & editing. **Matti J. Matheson:** Data curation, Formal analysis, Investigation, Methodology, Writing – original draft, Writing – review & editing. **Ashley J. Petersen:** Data curation, Formal analysis, Investigation, Methodology, Validation, Visualization, Writing – original draft, Writing – review & editing. **Adam Hansen:** Data curation, Formal analysis, Methodology, Writing – review & editing, Project administration. **Adam D. Block:** Data curation, Formal analysis, Investigation, Project administration, Writing – review & editing. **Corey Strong:** Data curation, Formal analysis, Writing – review & editing. **Annamarie Hill:** Data curation, Formal analysis, Writing – review & editing. **Meghan Kremer:** Data curation, Formal analysis, Writing – review & editing. **Ann J. Robertson:** Data curation, Formal analysis, Writing – review & editing. **Valmiki Maharaj:** Data curation, Formal analysis, Writing – review & editing. **Kamakshi Lakshminaryan:** Data curation, Formal analysis, Writing – review & editing. **Danni Li:** Data curation, Formal analysis, Writing – review & editing. **J. Neil Henderson:** Data curation, Formal analysis, Writing – review & editing. **Igor Nestrasil:** Data curation, Formal analysis, Writing – review & editing. **Christophe Lenglet:** Data curation, Formal analysis, Writing – review & editing. **William G. Mantyh:** Conceptualization, Data curation, Formal analysis, Funding acquisition, Investigation, Methodology, Project administration, Resources, Supervision, Validation, Visualization, Writing – original draft, Writing – review & editing.

## Ethical statement

Each participant (or their legally authorized representative if cognitive loss was to an extent that precluded informed consent) provided written informed consent to participate in this cross-sectional study. The Bois Forte Band of Chippewa partnered with the research team, approved this work through a signed, unanimously agreed-on tribal resolution, and approved the publication and public dissemination of this article. The University of Minnesota Institutional Review Board also approved this study.

## Declaration of competing interest

Rene Labounek reports no disclosures.

Matti Matheson reports no disclosures.

Ashley Petersen reports no disclosures.

Corey Strong Jr. reports no disclosures.

Annamarie Hill reports no disclosures.

Meghan Kremer reports no disclosures.

Ann Robertson reports no disclosures.

Adam Block reports no disclosures.

Adam Hansen reports no disclosures.

J. Neil Henderson reports compensation from Genentech and Roche for his role as Advisor on health equity and underrepresentation of Indigenous people in dementia research.

Valmiki Maharaj reports personal compensation for Pfizer Speakers Bureau (past activity: October 2024), consulting for BridgeBio Advisory Board participation (past activity: March 2025), and travel grants from Abbott Laboratories (past activity: October 2024).

Kamakshi Lakshminarayan reports compensation from Abbott Labs for service on adverse events committee related to device trials.

Igor Nestrasil reports no disclosures.

Christophe Lenglet reports no disclosures.

William G. Mantyh reports Personal compensation for consulting with Gerstman-Lehrman Group (current); Honoraria for Northwest Portland Area Indian Health Board (past activity: August 2024); Honoraria for NIH Neuroscience Grand Rounds (past activity: June 2024); Personal compensation as moderator of Regional Clinical Advisory Board meeting for gantenerumab with Genentech/Roche (past activity: November 2022); Personal compensation for educational videos Roon.com (past activity: May 2023); Personal compensation as a consultant with Lundbeck (past activity: December 2024).

## Data Availability

Data sharing must be approved by the Bois Forte Band of Chippewa. Investigators are asked to contact the corresponding author to initiate this request.
