# Supplementary material for: APOE ε4 and ischemic heart disease in American Indian/Indigenous tribal Elders
Source: Am Heart J Plus. 2025 Nov 4;60:100662. doi: 10.1016/j.ahjo.2025.100662 (PMC12639555; doi:10.1016/j.ahjo.2025.100662)
Supplement: Supplementary Table 1 — Comparison of participant characteristics of those included in the analysis vs. those excluded due to no blood sample, genotyping, or hemoglobin A1C (HbA1C). [file mmc1.docx]

**Supplementary Table 1.** Comparison of participant characteristics of those included in the analysis vs. those excluded due to no blood sample, genotyping, or hemoglobin A1C (HbA1C)

| **Characteristic** | **No Ischemic Heart Disease** | | | | **Ischemic Heart Disease** | | | |
| --- | --- | --- | --- | --- | --- | --- | --- | --- |
|  | **N** | **Excluded from the Analysis^1^**  **N = 101** | **Included in the Analysis^1^**  **N = 156** | **p-value^2^** | **N** | **Excluded from the Analysis^1^**  **N = 15** | **Included in the Analysis^1^**  **N = 25** | **p-value^2^** |
| **Age [years]** | 257 | 64 (59, 69) | 67 (61, 72) | 0.03 | 40 | 66 (63, 75) | 67 (63, 76) | 0.76 |
| **Female** | 257 | 71 (70%) | 112 (72%) | 0.80 | 40 | 7 (47%) | 14 (56%) | 0.57 |
| **Body Mass Index [kg/m^2^]** | 231 | 29 (25, 35) | 30 (26, 34) | 0.80 | 37 | 29.6 (26.2, 31.5) | 31.8 (26.1, 35.5) | 0.23 |
| **Tobacco Use** | 256 |  |  | 0.44 | 40 |  |  | 0.22 |
| Current |  | 46 (46%) | 66 (43%) |  |  | 7 (47%) | 9 (36%) |  |
| Former |  | 24 (24%) | 30 (19%) |  |  | 6 (40%) | 6 (24%) |  |
| Never |  | 31 (31%) | 59 (38%) |  |  | 2 (13%) | 10 (40%) |  |
| **Alcohol Use** | 256 |  |  | 0.86 | 40 |  |  | 0.91 |
| Current |  | 24 (24%) | 36 (23%) |  |  | 2 (13%) | 5 (20%) |  |
| Former |  | 39 (39%) | 65 (42%) |  |  | 8 (53%) | 11 (44%) |  |
| Never |  | 38 (38%) | 54 (35%) |  |  | 5 (33%) | 9 (36%) |  |
| **Participant Exercises** | 227 | 76 (78%) | 86 (67%) | 0.07 | 38 | 12 (80%) | 14 (61%) | 0.29 |
| **Self-Reported Hypertension** | 257 | 55 (54%) | 86 (55%) | 0.92 | 40 | 12 (80%) | 22 (88%) | 0.65 |
| **Self-Reported Hyperlipidemia** | 257 | 41 (41%) | 72 (46%) | 0.38 | 40 | 11 (73%) | 24 (96%) | 0.06 |
| **Self-Reported Chronic Kidney Disease** | 257 | 14 (14%) | 14 (9.0%) | 0.22 | 40 | 1 (6.7%) | 6 (24%) | 0.22 |
| **Statin use** | 257 | 27 (27%) | 39 (25%) | 0.76 | 40 | 8 (53%) | 18 (72%) | 0.23 |
| **Reason for Exclusion** | 257 |  |  |  | 40 |  |  |  |
| No Blood Sample |  | 91 (90%) | — |  |  | 13 (87%) | — |  |
| No Genotyping |  | 8 (7.9%) | — |  |  | 1 (6.7%) | — |  |
| No HbA1C from Blood |  | 2 (2.0%) | — |  |  | 1 (6.7%) | — |  |
| ^1^Median (IQR); n (%) | | | | | | | | |
| ^2^Wilcoxon rank sum test; Pearson's Chi-squared test; Fisher's exact test | | | | | | | | |
